# Ready for new waves: optimizing SARS-CoV-2 variants monitoring in pooled samples with droplet digital PCR

**DOI:** 10.3389/fpubh.2023.1340420

**Published:** 2024-01-12

**Authors:** Antonella Pacini, Franco Paredes, Sofia Heckel, Guadalupe Ibarra, Maria Victoria Petreli, Marilina Perez, Yanina Agnella, Laura Piskulic, Maria Belen Allasia, Luis Caprile, Alejandro Colaneri, Juliana Sesma

**Affiliations:** ^1^Molecular Biology Department, Hospital Provincial de Rosario, Rosario, Argentina; ^2^Instituto de Inmunología Clínica y Experimental de Rosario, CONICET, Rosario, Argentina; ^3^Facultad de Ciencias Bioquímicas y Farmacéuticas de Rosario, Universidad Nacional de Rosario, Rosario, Argentina; ^4^Facultad de Ciencias Veterinarias, Universidad Nacional de Rosario, Casilda, Argentina; ^5^Área Estadística y Procesamiento de Datos, Facultad de Ciencias Bioquímicas y Farmacéuticas, Universidad Nacional de Rosario, Rosario, Argentina; ^6^Consejo Nacional de Investigaciones Científicas y Técnicas, Rosario, Argentina; ^7^Facultad de Ciencias Médicas, Universidad Nacional de Rosario, Rosario, Argentina

**Keywords:** Pool testing, droplet digital PCR, COVID-19 pandemic, new variant outbreak, SARS-CoV-2 wave surveillance, Delta/Omicron tracking

## Abstract

**Introduction:**

The declaration of the end of the Public Health Emergency for COVID-19 on May 11th, 2023, has shifted the global focus led by WHO and CDC towards monitoring the evolution of SARS-CoV-2. Augmenting these international endeavors with local initiatives becomes crucial to not only track the emergence of new variants but also to understand their spread. We present a cost-effective digital PCR-based pooled sample testing methodology tailored for early variant surveillance.

**Methods:**

Using 1200 retrospective SARS-CoV-2 positive samples, either negative or positive for Delta or Omicron, we assessed the sensitivity and specificity of our detection strategy employing commercial TaqMan variant probes in a 1:9 ratio of variant-positive to variant-negative samples.

**Results:**

The study achieved 100% sensitivity and 99% specificity in 10-sample pools, with an Area Under the Curve (AUC) exceeding 0.998 in ROC curves, using distinct commercial TaqMan variant probes.

**Discussion:**

The employment of two separate TaqMan probes for both Delta and Omicron establishes dual validation routes, emphasizing the method’s robustness. Although we used known samples to model realistic emergence scenarios of the Delta and Omicron variants, our main objective is to demonstrate the versatility of this strategy to identify future variant appearances. The utilization of two divergent variants and distinct probes for each confirms the method’s independence from specific variants and probes. This flexibility ensures it can be tailored to recognize any subsequent variant emergence, given the availability of its sequence and a specific probe. Consequently, our approach stands as a robust tool for tracking and managing any new variant outbreak, reinforcing our global readiness against possible future SARS-CoV-2 waves.

## Introduction

The dynamic nature of COVID-19 pandemic is characterized by the emergence and succession of severe acute respiratory syndrome coronavirus 2 (SARS-CoV-2) variants, categorized as variants of concern (VOCs), variants of interest (VOIs), and, more recently, variants being monitored (VBM) (1–5).

As of this writing, the World Health Organization (WHO) recognizes Omicron (B.1.1.529 and descendant lineages) as the only VOC, while the Alpha (B1.1.7), Beta (B.1.351), Gamma (P1), and Delta (B.1.617.2) are considered VBM (1). The declaration of the end of the Public Health Emergency for COVID-19 on May 11th, 2023, has shifted the global focus led by WHO and CDC towards monitoring the evolution of SARS-CoV-2.

Traditional methods for tracking variants of SARS-CoV-2, such as Next-Generation Sequencing (NGS), provide definitive identification of mutations. NGS would be the method of choice when one seeks to monitor the emergence of new variants with unknown sequences. However, in the event of a wave of a new VOC, the timeliness of NGS falls short of the immediate demands for treatment decisions and for the surveillance required in the face of a quickly spreading virus (6, 7). Furthermore, the cost and infrastructure required for NGS are substantial, limiting its reach to countries with the necessary resources. In this context, more accessible and rapid methods would be needed. One such alternative is RT-PCR genotyping panels, which have enabled the quick identification of circulating SARS-CoV-2 variants (8, 9). However, these strategies primarily rely on individual testing, thereby escalating the time, labor, and costs involved.

Droplet digital PCR (ddPCR), an increasingly popular method for nucleic acid detection and quantification, offers an alternative approach (10–12). This technique compartmentalizes DNA molecules into thousands of droplets, amplifying them simultaneously, leading to several advantages, including decreased inhibitor concentration, increased relative sample concentration, and improved accuracy. The benefits of ddPCR have led to its widespread adoption in clinical research for the diagnosis of various diseases (13–16). Within our ddPCR process, two TaqMan probes with different fluorophores are utilized for amplification. These probes only differ in the sequence corresponding to the mutation of the variant under study. The probe with the HEX-fluorophore typically hybridizes specifically to the wild-type (WT) sequence, while the probe with the FAM fluorophore is specific for the sequence carrying the point mutation of interest. Our current digital PCR technology, featuring two channels of detection, supports the use of multiple probes, as evidenced in several studies (17, 18). Effective multiplexing is achieved by employing the same fluorophore for various probes, with distinct signal differentiation accomplished by adjusting the concentration of each probe. This allows for the separation of signal clusters in a two-dimensional plot, enhancing the versatility of digital PCR. Our system is designed with primers and probes that amplify a specific fragment size, typically between 60 to 100 base pairs. Thus, the size of the target marker does not affect the intensity of fluorescence in our assays. Rather, the fluorescence intensity within a positive droplet is predominantly influenced by the concentration of primers and probes used in the reaction. A higher concentration of these components generally results in stronger fluorescence signals, which is a crucial aspect for multiplexing applications. Therefore, by varying the concentrations of different probes, we can effectively multiplex without compromising the accuracy and efficiency of the assay.

In keeping with advancements in the field, newer versions of ddPCR technology now allow up to six channels to be read, significantly enhancing the analytical capabilities of ddPCR. This advancement enables the quantification of up to 12 targets in a single well, representing a substantial increase in throughput and efficiency for complex analyses, further broadening its application in research and diagnostics.

Following amplification, the fluorescence of each droplet is read, and the concentrations of mutant and WT molecules are determined using a Poisson distribution (19).

The utilization of pooling assays in diagnostic PCR offers significant advantages, including reduced costs, increased testing capacity, and shorter turnaround times, as multiple samples can be simultaneously analyzed in a single assay, providing an efficient and cost-effective approach to detect and monitor pathogens in diagnostics assays (20–22).

In this paper, we present a systematic variant detection approach that we evaluated on a total of 1,200 retrospective samples collected between August 2021 and May 2022 using commercially TaqMan variant probes. We analyzed the Delta and Omicron variants in pools of 10 SARS-CoV-2 positive samples, achieving a sensitivity of 100% and a specificity of 99% compared to individual samples.

During the initial phase of the Delta variant’s emergence, we evaluated our platform using a double-entry chart combinatorial pool-testing strategy on 41 SARS-CoV-2 positive samples. This approach efficiently reduced the number of samples needing ddPCR testing from 41 to 13. Upon identifying positive intersections of rows and columns, we performed individual RT-PCR tests on the intersecting samples and were able to detect all three that carried the Delta variant. This led to notable savings in both reagents and time.

While our study focused on known, retrospective samples and targeted SARS-CoV-2 variants that may no longer be relevant for current testing, this was a necessary step to fine-tune the method. The true intent behind this work is to ensure that, in the future, this methodology can be applied to monitor the circulation of any newly emerging variant. The ddPCR pooling strategy is especially effective for detecting these SARS-CoV-2 variants when they first begin to circulate and their incidence is very low, allowing for pooled testing. In this landscape, both NGS and ddPCR techniques can complement each other. While NGS stands out in the identification of new mutations, ddPCR excels in tracking their distribution, making it an invaluable tool for rapid response to any rapidly evolving pathogens with pandemic potential.

## Materials and methods

### Sample collection

Nasopharyngeal samples were collected between August 2021 and May 2022, mixed with 2 mL saline solution and stored at 4°C until extraction. In Biosafety Level 2 containment, the virus was inactivated and then extracted automatically using the Applied Biosystems^™^ MagMAX^™^ kit and the Thermo Scientific^™^ KingFisher^™^ purification system.

### Individual RT-qPCR test

RT-qPCR was performed in the clinical laboratory to detect the presence of SARS-CoV-2 RNA with the PerkinElmer SARS-CoV-2 RT-qPCR Reagent kit^®^, following manufacturer instructions. We received de-identified positive samples with a code. SARS-CoV-2 positive samples were genotyped by RT-PCR to detect the variants of concern (Delta and Omicron). For that, probes P681H.CCT.CAT, L452R.CTG.CGG, Q954H.CAA.CAT and P681R.CCT.CGT (ThermoFisher Scientific^®^) and CoV_B1429_L452R (Biorad^®^) were used. At the time of assay, the spike protein mutation P681R was used to identify and distinguish the Delta variant and L452R was specific for Delta and Epsilon. On the other hand, Q954H was specific for Omicron while P681H was specific for Omicron and Alpha variants.

### Pooling preparation

To confirm that the samples employed for pool creation were devoid of the variants under study, we utilized samples collected prior to the emergence of these variants. We designated these samples, which tested positive for SARS-CoV-2 but negative for the specific variant in study, as “WT-SARS-CoV-2 samples.” The SARS-CoV-2 sample carrying the mutation in study was designated VARIANT-SARS-CoV-2 (Va-SARS-CoV-2) sample. For the construction of sample pools, we combined one Va-SARS-CoV-2 sample with nine WT-SARS-CoV-2 samples.

### Primer/probe thermal gradient optimization

Optimus pool size and dilution were used as a template to assess the optimal annealing temperature of the individual primer/probes. The standard ddPCR cycling program was modified by replacing the annealing temperature step with a thermal gradient from 55°C to 60°C for 1 min extension time. These experiments were performed with P681H and Q954H from Thermo Fisher Scientific^®^ and L452R from Thermo Fisher Scientific^®^ and Biorad^®^.

### Pooled ddPCR test

One-Step RT-ddPCR Advanced Kit for Probes (BioRad) was used for RT-ddPCR according to the manufacturer’s recommendations. 5,5 μL of pooled samples were added to 16,5 ul of master mix with variants/WT primers and probes according to the manufacturer’s recommendations. VOCs probes (P681H, L452R, Q954H, and P681R) were labeled with carboxyfluorescein (FAM), WT probes were labeled with HEX fluorophore. RT-ddPCR reactions were set up in 96-well ddPCR plates. Plates were covered with a pierceable foil heat seal, sealed using a PX1 PCR Plate Sealer (BioRad), mixed by vortexing, and centrifuged before droplet generation. Droplet generation was done in the QX200 AUTO DG (BioRad), which dispensed droplets into a new 96-well plate. Then, plates were heat-sealed with a pierceable foil and thermocycled on a C1000 touch thermal cycler (BioRad) as follows: 50°C for 50 min; 95°C for 10 min; 5 cycles of 94°C for 15 s and 55°C for 50s, 35 cycles of 94°C for 10 s and 55°C for 45 s, 98°C for 10 min, followed by a 25 min hold at 4°C for droplet stabilization. Droplets were then read on a QX200 Droplet Reader (BioRad) set up to read FAM and HEX channels and analyzed using the QuantaSoft^™^ Analysis Pro 1.0.596 software. The software performed the automated droplet count for each case, which was then manually reviewed to determine whether positive droplets fell outside gating parameters. Besides the cut-off determined and according to manufacturer instructions, a minimum number of accepted droplets is required to ensure optimal detection in the analysis: positive assays must have at least 6,000 accepted droplets, negative ones must have 10,000 or more (23).

### Statistical analysis

The overall performance of the new technique was evaluated by calculating the area under the Receiver Operator Characteristics (ROC) curve and its 95% confidence interval using the method described by Hanley and McNeil (24). The best cut-off point was selected using the Youden Index (25), with a priority for the test to have a 100% sensitivity. The estimates of sensitivity and specificity were determined using a 95% confidence interval for previously established cut-off values.

Data were processed using R. Among other packages, pROC and OptimalCutpoints were utilized.

Analysis of the ddPCR data was performed with QuantaSoft analysis software v.1.7.4.0917 (Bio-Rad) to calculate the concentration of the targets.

### Ethical approval

The study protocol was approved on December 10th, 2020 by the Bioethics Committee of Universidad Nacional de Rosario, Facultad de Ciencias Médicas (resolution: 3733/2022). Signed informed consent was not necessary as de-identified samples of SARS-CoV-2 tests by HOSPITAL PROVINCIAL DE ROSARIO were used. This study was conducted in accordance with the principles of the 1964 Declaration of Helsinki.

## Results

### Assay optimization: pool size and thermal gradient

While our previous work demonstrated the ability to detect a SARS-CoV-2 positive sample within a 34-sample pool (26), identifying specific Va-SARS-CoV-2 sequences among SARS-CoV-2 positive samples proves to be more complex. This complexity arises because, in the former case, we were seeking a specific sequence within a vast array of DNA without similar sequences. In contrast, now we face the added challenge of differentiating between the target sequence and the wild-type sequence, which differ by only a single nucleotide.

**Figure 1:**
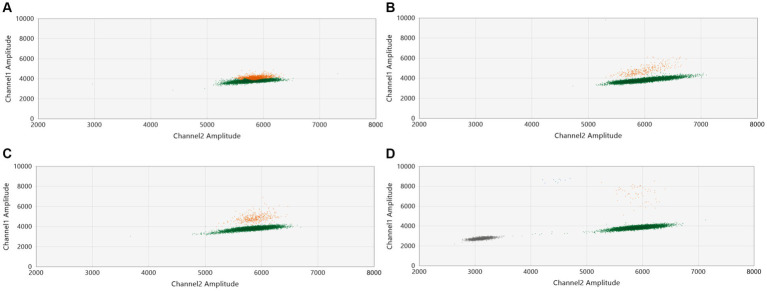
Assay optimization: influence of sample dilution on assay performance. A Delta sample (CT = 27) was combined with nine WT-SARS-CoV-2 samples (CT values ranging from 14 to 20). The combined pool was then analyzed by ddPCR with the L452R probe (Catalogue # CVAAAAD). We examined the distribution of droplet populations across various dilution conditions with water: **(A)** undiluted, **(B)** 1/2 dilution, **(C)** 1/4 dilution, and **(D)** 1/30 dilution. The diagrams illustrate the variation in droplet distribution under each dilution condition.

**Figure 2:**
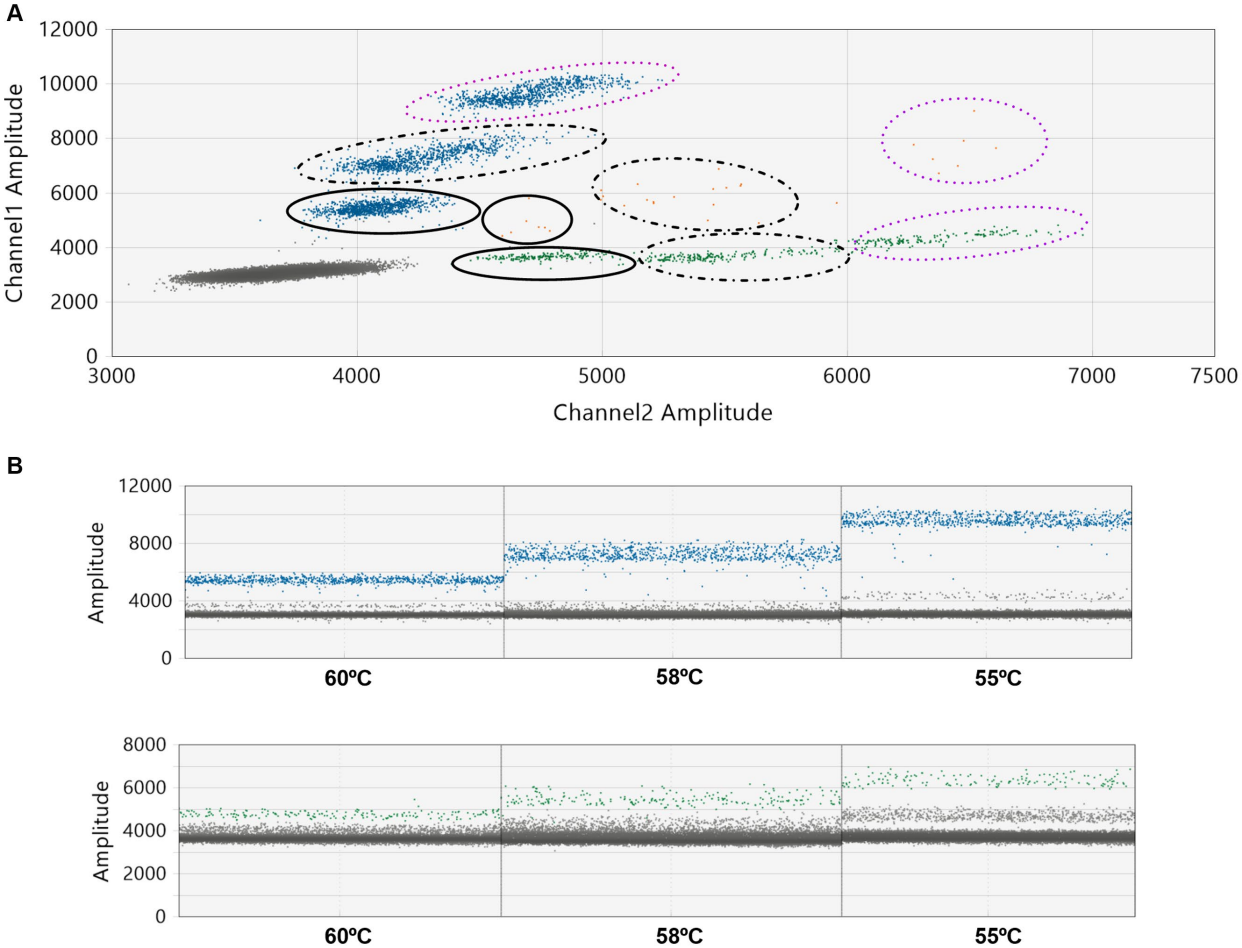
Assay optimization: impact of thermal gradient on annealing temperature. A Delta sample (CT = 24) was combined with nine WT-SARS-CoV-2 samples (CT values ranging from 14 to 20). This pooled sample was then analyzed by ddPCR with the L452R probe (CoV_B1429_L452R Biorad^®^). Assays were conducted at three different annealing temperatures: 55°C, 58°C, and 60°C. **(A)** A 2-D plot displaying the distribution of the droplet populations at each of the three annealing temperatures. The smooth line represents 60°C, the dashed line represents 58°C, and the purple dotted line represents 55°C. **(B)** 1-D plots of the ddPCR reactions at different temperatures, with blue representing positive droplets for the variant, green for the WT, and gray for negative droplets.

In our initial tests, we purposefully mixed one Delta sample (with a CT value of 27) with nine WT-SARS-CoV-2 samples. We intentionally chose a sample with a high CT value, which holds clinical relevance, to challenge the system’s capability in detecting variants at a low concentration (27). Assay conditions were then modified for optimization. As depicted in Figures 1A–D the dilution of the pool with water (1/2, 1/4, and 1/30) and the reduction of the annealing temperature expanded the separation between the WT and mutant droplet clouds (Figures 2A,B). This increase in the separation of the clouds in a graph was already described by the manufacturer (28). The optimal condition was found to be a 1/30 pool dilution in water combined with an annealing temperature of 55°C.

### Specificity and sensitivity assessment

Recognizing the competitive amplification environment where the mutant sequence is outnumbered by the predominance of WT sequence, it was crucial to establish conditions enhancing the method’s specificity. We assessed the specificity of our method in accordance with the guidelines of the European Pharmacopoeia (29). We obtained 1,000 WT SARS-CoV-2 samples that predate the Delta variants’ emergence. These samples were divided into 100 pools for analysis, and each pool was examined by ddPCR with the 4 probes named in Table 1. Each probe had to be run independently to ascertain their distinct cutoff points. The cut-off points were the maximum number of positive drops tolerated in a negative sample.

**Table 1:** Details of sensitivity, specificity, and area under the curve (AUC) for all four probes used for SARS-CoV-2 variant detection.

Variant	Amino acid change	Cut off number	AUC	Specificity *n* = 100	Sensitivity *n* = 24
Omicron	P681H	4,6	0.999	99%	100%
Delta	P681R	2,8	0.998	99%	100%
Delta	L452R	2,75	1	100%	100%
Omicron	Q954H	7,45	1	100%	100%

Evaluating the sensitivity of our platform presented a unique challenge due to the need to detect a specific variant sequence amidst a majority of WT-SARS-CoV-2 samples. To accurately measure this sensitivity, we designed our tests according to the guidelines provided by the European Pharmacopoeia (27). Our sensitivity analysis was initiated with a Delta SARS-CoV-2 sample (CT = 27) combined with nine WT SARS-CoV-2 samples (with CT values ranging from 14 to 20). This high-CT Delta sample was purposefully chosen to test the system’s capacity to detect a low-concentration variant amid a higher-concentration of WT SARS-CoV-2 samples. To ensure the robustness of our findings, each measurement was repeated six times across four independent experiments, resulting in a total of 24 repetitions for each probe. This process was conducted separately for each of the two Delta variant-specific probes. A similar study design was applied for the two probes specific to the Omicron variant. Table 1 presents the results. The sensitivity at the best cut-off value was 100% for all probes, while the specificity was 100% for the Q954H and L452R probes, and 99% for the P681R and P681H probes.

**Figure 3:**
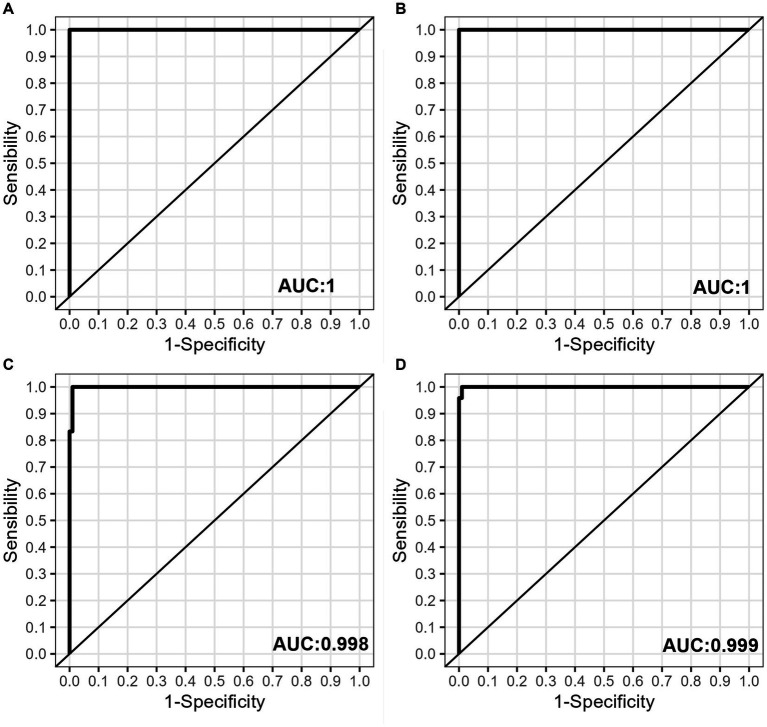
Sensitivity and specificity assessment. Receiver operating characteristic (ROC) curves are illustrated for the following probes: **(A)** L452R, **(B)** Q954H, **(C)** P681R, and **(D)** P681H. These curves were derived from the analysis of pooled samples consisting of one Va-SARS-CoV-2 sample (CT = 27) and nine WT-SARS-CoV-2 samples (CT values ranging from 14 to 25).

The Receiver Operating Characteristic (ROC) curves were used to analyze the specificity and sensitivity of the four different probes (Figures 3A–D). Table 1 shows that the areas under the curve (AUC) were 1 for the Q954H and L452R probes, and above 0.998 for the P681R and P681H probes.

In regards to the P681R and P681H probes, we prioritized having 100% sensitivity over specificity as we did not want to miss any positive sample. Conversely, if a pool tested false positive, this error could be eliminated by de-pooling and individually testing each sample via RT-PCR.

### Clinical case study

We had the opportunity to evaluate our platform during the emergence of the Delta variant wave from September 17th to 22nd, 2021. At that time, the incidence of Delta cases in our country was still quite low, representing about less than 2% of total cases (30). We selected 41 leftover SARS-CoV-2 positive samples from that week and applied them to a double-entry chart combinatorial pool-testing strategy, also known as matrix or grid testing (20). This approach entails testing each sample twice in a grid format, which facilitates the cross-matching of results to accurately identify positive samples. The methodology is visually represented in Table 2. As the incidence was 2%, pools were assembled with either six or seven samples each.

**Table 2:** Double-entry chart with analyzed samples for the real case.

	POOL A	POOL B	POOL C	POOL D	POOL E	POOL F
POOL 1	17,664	17,673	18,587	18,595	21,675	22,627
POOL 2	17,667	17,674	18,588	20,597	21,525	22,628
POOL 3	17,668	18,592	18,589	20,598	21,671	22,629
POOL 4	17,669	20,567	18,590	20,599	21,672	22,630
POOL 5	17,670	19,509	18,591	20,600	21,673	22,631
POOL 6	17,671	19,510	18,593	20,601	22,657	22,616
POOL 7	17,672	19,511	18,594	21,674	22,626	

Samples that were used for the preparation of 13 pools are shown in this table (numbers in the cells are internal codes of identification of patients used in the hospital). Pools 1–7 were prepared with samples described under each line, and pools A-F were prepared with samples described following each column. Considering this distribution and preparation of pools, each sample was tested twice, once in pool 1–7 and once in pool A-F. In dark gray we show the “putative positive samples” to be retested by RT-qPCR after finding that pools 5,6,7, B and E were positives by ddPCR.

Table 2 shows that pools B, E, 5, 6, and 7 returned positive results for the Delta variant (light gray). The cells located at the intersection of these columns and rows, indicated in dark gray, were designated as “putative positive samples.” We proceeded to “deconstruct” these five pools and individually re-tested the six dark gray samples using RT-PCR. This process resulted in the confirmation of samples 19–509, 19–510, and 22–626 as Delta variant positives.

It is important to highlight that this approach effectively reduced the number of samples requiring ddPCR testing from 41 down to 13 (plus an additional 6 samples individually tested by RT-PCR). Furthermore, this method ensured each sample was tested twice: once in column pools and once in row pools.

We employed this technology briefly. Within just one week, the prevalence of the delta variant in our country jumped from 2% to over 10%. Given this rapid increase and the subsequent high prevalence, pooling samples became impractical, as the majority were expected to test positive for the variant (30).

## Conclusion

Despite the declaration of the end of the Public Health Emergency for COVID-19 on May 11th, 2023, national efforts to monitor the emergence and spread of new variants continue. While these strategies may vary from country to country, they generally involve genomic surveillance of a percentage of patients, travelers, and wastewater samples (1).

Although next-generation sequencing remains the primary method for detecting and tracing new SARS-CoV-2 variants, there are scenarios where its inherent challenges – such as cost, extended turnaround times, and the need for specialized personnel – could benefit from complementary methodologies (6, 7). Techniques like Sanger sequencing and RT-PCR genotyping using single-nucleotide polymorphism (SNP) can serve as supplementary tools, providing faster and more cost-effective solutions in certain contexts (8, 31, 32). Digital PCR is a sophistication of the traditional RT-PCR that has higher sensitivity to detect a low copy number mutation (33). Additionally, pooling samples with ddPCR offers the significant benefit of reducing reagent costs. The extent of these savings is influenced by several factors, including the positivity rate, which in turn guides the optimal group size, as well as the inherent cost of ddPCR testing. When comparing the costs, if ddPCR is approximately twice as expensive as RT-PCR, pooling 1,000 samples in groups of 10 with a positivity rate of 2% can result in reagent cost reductions of at least 50%.

Our study presents a robust and flexible platform for the detection of specific SARS-CoV-2 variants amidst a backdrop of 10 WT-SARS-CoV-2 samples. We have successfully adapted and optimized our earlier SARS-CoV-2 detection methodology (26) leveraging a ddPCR pooling strategy, to identify two different variants, Delta and Omicron.

A pivotal strength of our platform lies in its inherent adaptability. It is designed to be modifiable with minimal alterations needed to detect future emerging variants. Through the strategic selection of two distinct TaqMan probes for each variant, we established two independent methods for each variant detection, enhancing the reliability and versatility of our platform.

Our platform’s sensitivity and specificity were carefully evaluated following the European Pharmacopoeia. We demonstrated 100% sensitivity at the best cut-off value for all probes, with specificity ranging from 99% to 100% across the four probes used. The real-world efficacy of our platform was validated during the emergence of the Delta variant, accurately identifying Delta-positive samples within the tested pools.

In conclusion, we have developed a robust, sensitive, and flexible platform capable of accurately identifying specific SARS-CoV-2 variants within predominantly WT-SARS-CoV-2 sample pools. Moreover, the underlying principles and techniques of this platform hold significant potential for broader applications. It is not only adaptable for detecting variants from other viruses beyond SARS-CoV-2 but also versatile enough to work effectively across various sample matrices. Digital PCR’s ability to detect different types of viruses, including respiratory pathogens and others like Ebola and Dengue, in blood samples, has been validated (34, 35). For instance, our work on the detection of SARS-CoV-2 in saliva (26), and the effectiveness of digital PCR in other matrices such as urine and feces for SARS-CoV-2 (36) and bacteria in blood (34), underlines the flexibility of this technology in a variety of diagnostic scenarios.

The adaptability and precision of this platform may thus contribute profoundly not only to the current fight against COVID-19, but also to future endeavors in viral epidemiology and public health. By simply modifying the targeted genetic sequences, our methodology could serve as a versatile foundation for rapid response to new viral threats, enhancing our capabilities in global health surveillance and response.

## Data availability statement

The datasets presented in this study can be found in online repositories. The names of the repository/repositories and accession number(s) can be found in the following link: https://doi.org/10.6084/m9.figshare.24915639.

## Ethics statement

The studies involving humans were approved by COMITÉ DE BIOSEGURIDAD, Facultad de Ciencias Medicas, Universidad Nacional de Rosario. The studies were conducted in accordance with the local legislation and institutional requirements. The human samples used in this study were acquired from remnants from SARS-CoV-2 tests conducted at the Hospital Provincial de Rosario. Signed informed consent was waived as patient information was anonymized and de-identified prior to analysis. This study was carried out in accordance with the principles of the 1964 Declaration of Helsinki. Written informed consent for participation was not required from the participants or the participants’ legal guardians/next of kin in accordance with the national legislation and institutional requirements.

## Author contributions

AP: Conceptualization, Data curation, Formal analysis, Investigation, Methodology, Writing – original draft, Writing – review & editing. FP: Conceptualization, Data curation, Formal analysis, Investigation, Methodology, Writing – original draft, Writing – review & editing. SH: Conceptualization, Data curation, Formal analysis, Investigation, Methodology, Writing – original draft. GI: Conceptualization, Data curation, Formal analysis, Investigation, Methodology, Writing – original draft. MVP: Conceptualization, Formal analysis, Investigation, Methodology, Writing – original draft. MaP: Data curation, Formal analysis, Investigation, Methodology, Writing – original draft. YA: Investigation, Methodology, Writing – review & editing. LP: Formal analysis, Writing – review & editing. MA: Formal analysis, Writing – review & editing. LC: Investigation, Writing – original draft. AC: Conceptualization, Funding acquisition, Investigation, Resources, Writing – review & editing. JS: Conceptualization, Formal analysis, Investigation, Methodology, Project administration, Resources, Supervision, Writing – original draft, Writing – review & editing.
